# Establish Global Nutrition Research Strategies: The Meeting Report of the First SIOP Nutrition Research Forum

**DOI:** 10.3390/nu18071112

**Published:** 2026-03-30

**Authors:** Rajul M. Gala, Judy Schoeman, Raquel Revuelta Iniesta, Mirjam van den Brink, Amy L. Lovell, Minke H. W. Huibers

**Affiliations:** 1 Division of Paediatric Oncology, Tata Memorial Centre, Mumbai 400 012, India; rajulgala1@gmail.com; 2 Division of Paediatric Oncology and Haematology, Department of Paediatrics and Child Health, Faculty of Medicine and Health Sciences, University of Pretoria, Pretoria 0001, South Africa; judy.schoeman@up.ac.za; 3 Department of Pediatrics and Child Health, Faculty of Medicine and Health Science, Stellenbosch University, Stellenbosch 7505, South Africa; m.h.w.huibers-2@prinsesmaximacentrum.nl; 4 Public Health and Sports Sciences, Faculty of Life and Health Sciences, Medical School, University of Exeter, Exeter EX2 4TE, UK; 5 Child Life and Health, University of Edinburgh, Edinburgh EH16 4TJ, UK; 6 Princess Maxima Center for Pediatric Oncology, 3584 CS Utrecht, The Netherlands; m.vandenbrink-9@prinsesmaximacentrum.nl; 7 Department of Nutrition and Dietetics, Faculty of Medical and Health Sciences, The University of Auckland, Auckland 1023, New Zealand; a.lovell@auckland.ac.nz; 8 Starship Blood and Cancer Centre, Starship Child Health, Te Whatu Ora, Te Toka Tumai, Auckland 1142, New Zealand; 9 Global Child Health Group, Amsterdam University Centre, 1100 DD Amsterdam, The Netherlands

**Keywords:** pediatric oncology, cancer in children, nutrition, nutrition research forum, meeting report

## Abstract

Despite strong evidence linking nutritional status to treatment efficacy and survival in pediatric cancer, significant knowledge gaps and practice variation persist globally. On 24th October 2025, the International Society of Paediatric Oncology (SIOP) Nutrition Network, in collaboration with Prinsess Máxima Center for Paediatric Oncology and the International Initiative for Pediatrics and Nutrition (IIPAN), convened the first global SIOP Nutrition Network Research Forum. The forum brought together 54 international experts from high-income countries and low- and middle-income countries to define global nutrition research strategies for pediatric oncology. The forum addressed six emerging domains: body composition and treatment outcomes; microbiome, micronutrient status and metabolic health; prehabilitation and rehabilitation strategies; validation of nutritional assessment tools, guideline development for high-income settings; insights from international multicentric research initiatives—the International Atomic Energy Agency (IAEA), SIOP Nutrition Network, the Adapted Resource and Implementation Application (ARIA) guide nutrition portal, the International Collaboration on Nutrition in Cancer (ICONIC) WHO knowledge portal; and IIPAN and the World Cancer Research Fund (WCRF) for funding strategies. Delegates identified three priority working groups, namely prehabilitation optimization, pharmacokinetics, and advocacy, with each outlining collaborative nutrition research priorities for the next five years. This forum represents a critical point in pediatric oncology nutrition research as it established the first coordinated and internationally endorsed roadmap to bridge gaps in cancer care and ensure standard nutrition care worldwide. The research priorities and collaborations will help in creating evidence to improve cancer treatment and survival rate for children globally.

## 1. Introduction

Malnutrition has remained a critical challenge in pediatric oncology globally, with significant disparities in nutritional care capacity between high-income countries (HICs) and low- and middle-income countries (LMICs) [1]. In LMICs most children diagnosed with cancer live in poverty, experience food insecurity at home, and are undernourished at diagnosis, leaving them vulnerable for infection and treatment toxicity [2]. It is known that children diagnosed in LMICs have lower survival rates due to inaccessible treatment, treatment abandonment, and a lack of professionals [3], whereas HICs have specialists dedicated to nutrition and specialized nutritional assessment tools (e.g., bioelectrical impedance analysis (BIA)) available to determine body composition with targeted nutritional intervention, including both commercial specialized feeds and sterile nutrient solutions to target undernutrition and/or complex nutritional support needs [3]. The establishment of the Nutrition Network Research Forum reflects the growing recognition of nutrition as central to improving outcomes for children and young people (CYP) with cancer worldwide. The Forum has aimed to bring together leading researchers, dietitians, clinicians and advocates to synthesize current knowledge, identify evidence gaps, and establish collaborative priorities for advancing pediatric oncology nutrition as both a clinical practice and a research discipline.

On 24 October 2025, the International Society of Paediatric Oncology (SIOP) Nutrition Network, together with Prinses Máxima Center (Utrecht, The Netherlands) and the International Initiative for Pediatrics and Nutrition (IIPAN), organized the first SIOP Nutrition Network Research Forum held in Princess Máxima Center (Utrecht, The Netherlands). The agenda was planned by reviewing recent research on nutrition and identifying experts in those areas. Speakers in these fields were invited to present a topic, as well as delegates in the field of the forum, within the planned agenda to ensure interested parties attend. (Supplementary Table S1). Limited spaces were kept for registrations via advertisements through the SIOP channels, LinkedIn, and colleagues to also ensure attendance of non-SIOP members. The forum was attended by a total of 54 delegates from 20 countries, including 37 dietitians/nutritionists, eight pediatric oncologists, four physiotherapists, two research assistants, one nurse, one pharmacist, and a representative of the childhood cancer research fund. Scholarships were available for 13 delegates, for which support from SIOP has been acknowledged.

Delegates were welcomed to the forum by the co-chairs of the SIOP Nutrition Network, Dr Minke Huibers and Dr Judy Schoeman. They provided a brief overview of the Nutrition Network’s work and outlined the forum’s goals, namely, to form collaborative research and action priorities for the next five years. The invited speakers covered a broad range of priority topics in pediatric oncology nutrition by focusing on six emerging domains: the role of body composition in treatment outcomes and chemotherapy pharmacokinetic; microbiome, micronutrient status, and metabolic health; prehabilitation and rehabilitation strategies to integrate nutrition across the cancer care pathway; advances in nutritional assessment methodologies and validation of nutritional assessment tools, guideline development for high-income settings; insights from global research infrastructure with strategies for building international communities of practice—the International Atomic Energy Agency (IAEA), SIOP Nutrition Network, the Adapted Resource and Implementation Application (ARIA) guide nutrition portal, the International Collaboration on Nutrition in Cancer (ICONIC) WHO knowledge portal, IIPAN, and the World Cancer Research Fund (WCRF) for funding strategies. The forum concluded with three roundtable discussions that formed the three priorities for the following five years. The speakers’ summaries and the content of the forum meeting are discussed in the following sections.

## 2. Body Composition and Treatment Outcomes

### 2.1. Impact on Pharmacology and Drug Dosing

Prof Gareth Veal (UK) discussed a recently published systematic review and the presentation highlighted that most anti-cancer drugs were dosed using crude metrics based on adult data, despite clear differences in drug metabolism among children with varying nutritional states [1].

A rigorous analysis of the available research identified within this systematic review showed just 18 relevant studies for 12 drugs, revealing that: (1) malnutrition was suggested to increase doxorubicin and methotrexate exposure as these patients cleared the drug less efficiently [4,5]; (2) obesity raised the drug clearance for mercaptopurine, potentially lowering its effectiveness, and likely affected other chemotherapy agents as well [6]; (3) inconsistencies in defining and measuring nutritional status and the dosing practices across studies limited clinical application [1]; and (4) standardized prospective research is urgently needed to safely individualize dosing and improve outcomes for malnourished and obese children with cancer.

The systematic review revealed a critical knowledge gap, as no consensus or recommendations could be drawn due to the limited number of studies (only 18 available) despite theoretical understanding of how nutritional status affects drug disposition—empirical evidence for commonly used pediatric anti-cancer drugs has remained severely limited. The narrow therapeutic window of cytotoxic agents—where the margin between therapeutic response and excessive toxicity is small—has made this knowledge gap particularly concerning [1]. This presentation underscored that integrating nutritional status into pediatric chemotherapy protocols represented a major opportunity for improving both safety and efficacy in cancer care. Secondly, 90% of the evidence originated from HICs, with limited inclusion of severe malnourished children. Thirdly, it highlighted the current gap of evidence-informed practice incorporation undernutrition into treatment protocols.

### 2.2. Body Composition Assessment in Global Research

Dr Alexia Murphy-Alford (Austria) reported on the International Atomic Energy Agency (IAEA) nutrition work centering on stable isotope methodologies and clinical nuclear imaging techniques that have provided gold-standard assessments of body composition and energy metabolism. Key methodologies that have formed the foundation of this work include: deuterium dilution for body composition assessment, which involves a simple, non-invasive procedure using stable isotopes to measure total body water, fat, and fat-free mass [7]; doubly labeled water, which is the only validated method for measuring free-living energy expenditure, enabling an accurate assessment of metabolic demands during normal daily activities over 14 days [8]; and Dual-energy X-ray Absorptiometry (DEXA) scans and computed tomography (CT) imaging, which are used for bone density, body composition, and sarcopenia diagnoses in cancer patients [9].

Dr Murphy-Alford highlighted the Rays of Hope Initiative, which is a global program led by the IAEA that aimed to reduce cancer-related mortality by increasing access to safe and secure radiotherapy and diagnostic imaging worldwide. This initiative has directly addressed the identified gaps by integrating advanced nuclear techniques, such as deuterium dilution and doubly labeled water, to provide the high-precision data necessary for personalizing nutritional care. By utilizing these assessments, the program has allowed for the accurate measurement of metabolic demands and body composition changes that are critical for optimizing chemotherapy dosing and improving treatment tolerance in children with cancer. Working in partnership with national governments and international stakeholders, the initiative has focused on strengthening radiation safety, nuclear security legislation, and clinical infrastructure, prioritizing countries with the greatest unmet needs. A key strategic objective of Rays of Hope is the integration of nutrition into cancer care capacity building, achieved through collaborations with leading institutions such as MD Anderson Cancer Center and the Princess Máxima Center for Pediatric Oncology [10].

As part of this effort, the IAEA launched the multi-country research project “*Body Composition Changes and Clinical Outcomes in Children with Cancer*” in 2019. The objectives of this program were to report individual country results aligned to local research priorities, as well as to produce pooled analyses on body composition at diagnosis, validate simpler and more feasible assessment techniques such as BIA, and develop new BIA prediction equations. This prospective study involved nine countries (*Mexico, Jamaica, South Africa, Morocco, Russia, India, Malaysia, Thailand and the Philippines*) with standardized assessments at diagnosis and across four to five follow-up time points over 24–36 months. Data collection encompassed anthropometry, body composition (*using deuterium dilution, BIA and DEXA*), energy expenditure (*doubly labeled water and indirect calorimetry*), dietary intake, physical activity, functional capacity, quality of life, and clinical outcomes. This project was completed in December 2025, and the publications are under preparation.

Collectively, this body of work demonstrated that advanced nuclear and imaging techniques can be successfully implemented in LMICs to generate high-quality nutritional data in pediatric oncology. Through capacity building, standardized protocols, international collaboration, and strategic partnerships, the IAEA initiative bridged the gap between cutting-edge technology and equitable global access, advancing both the science and clinical practice of pediatric oncology nutrition [10].

### 2.3. Impact of Body Composition on Acquired Immuno-Deficiency Secondary to Malnutrition and Post-Discharge

Dr Wieger Voskuil (the Netherlands) presented on the burden and clinical implications of malnutrition in children, highlighting that approximately 45% of deaths in children under five years of age globally were due to being undernourished, with the highest prevalence occurring in LMICs [11]. He emphasized that certain pediatric populations were disproportionately affected, including children with cancer, who can face an elevated risk of adverse outcomes secondary to malnutrition. Vulnerability was particularly pronounced among children with poor anthropometric status, those experiencing acute illness, and children under two years of age, with emerging challenges such as climate change and food insecurity further exacerbating nutritional risk and inequities [12].

Secondly, Dr Voskuil highlighted that nutritional vulnerability was not confided as a single point in the care pathway that spans pre-hospitals, hospital admission, and up to six months following discharge, with both baseline nutritional status and disease severity playing a critical role in determining clinical outcomes [13]. Despite its clear relevance to prognosis and quality of life, this period of heightened vulnerability has remained insufficiently characterized and poorly addressed in current care models. Drawing on the WHO Integrated Management illness framework, he noted that the severity of illness, rather than etiology alone, was often the strongest predictor of adverse outcomes [14].

This highlighted the need for improved risk stratification across the pediatric patient journey, including the use of simple, scalable clinical parameters, the implementation of tailored and parent-inclusive discharge planning, and the development of post-discharge digital tools to enhance parental knowledge, monitoring, and informed decision-making.

## 3. Microbiome, Micronutrients, and Metabolic Health

### 3.1. Prebiotics, Probiotics, and Postbiotics in Cancer

Mr Adrian Maryniak (Poland) presented an overview of the emerging roles of prebiotics, probiotics, and postbiotics in cancer treatment, with a focus on their potential applications and limitations in oncology.

*Prebiotics*, such as inulin, can act by selectively stimulating beneficial gut microorganisms and have shown potential in enhancing chemotherapy efficacy and reducing cancer metastasis in leukemia models [15,16].

*Probiotics*, defined as live microorganisms administered in adequate amounts, are naturally present in fermented foods and have been shown to reduce chemotherapy-related gastrointestinal toxicity, modulate the immune function, and lower infection risks in patients with cancer [17]; however, their routine use in oncology settings has remained limited.

*Postbiotics*, which include inactivated microorganisms and their bioactive components such as short-chain fatty acids and vitamins (e.g., *folic acid*), have demonstrated anti-cancer activities, including apoptosis in leukemia cells, and may potentially reduce childhood cancer risk when supplemented during pregnancy [18].

Collectively, biotics can exert their effects through modulation of the gut microbiota, which can play a critical role in cancer development and treatment responses by modulating immune regulation, host metabolism, and the tumor microenvironment. Variations in microbial composition have been associated with different treatment efficacy, supporting the concept that personalized microbiota modulation could enhance cancer treatment [19,20]. However, clinical translation has been challenged by the lack of standardization in strain selection, dosing and timing, substantial inter-individual variability in responses, and potential safety concerns such as bacteremia in immuno-compromised individuals. A systematic review concluded that the risk for probiotic-associated infections or other adverse events sustained could not be ruled out due to heterogeneity of malignancies and/or the treatment regimens delivered. Subgroup analysis was not possible, particularly in children, so more research is needed in this field [21].

Future research directions are therefore expected to focus on tailored-based microbiota interventions, including the development of engineered bacteria therapies designed to selectively target tumor pathways [20,22,23].

### 3.2. Microbiome in Cancer Patients: What Do We Know?

Prof Elena Ladas (USA) highlighted key research on the gut microbiome’s impact in pediatric cancer, focusing on two main areas: administration of probiotic therapy among CYP undergoing hematopoietic stem cell transplantation (HSCT); and the association of microbial signatures and obesity in CYP with acute lymphoblastic leukemia (ALL).

In 2025, Ladas et al. demonstrated that the administration of *Lactobacillus plantarum299v (LBP299v)* in children undergoing conditioning therapy for HSCT was safe, leading to changes in microbial diversity; however, clinical benefits were not observed [24]. *LBP299v* did not reduce the incidence or severity of acute gastrointestinal graft-versus-host disease (aGvHD). The authors hypothesized that variable colonization, low dosing, single-strain administration, or a reduction in the incidence of aGvHD may explain the study’s findings. Other applications of microbiomes may be apparent in children with ALL who were at risk for the development of obesity. The authors hypothesized that perturbations in the microbiome, either due to prolonged exposure to antibiotic therapy or the treatment itself, may predispose children to develop obesity. In their paper published in *Leukemia Research*, distinct microbial signatures were associated with children who maintained a healthy weight compared to those developing obesity [25].

Additional questions have remained as to the temporal association of fluctuations in the microbiome and nutritional outcomes. Larger studies among children with varying exposures are needed to better understand microbiome–host interactions, optimize probiotic strategies, and understand the association between diet, the microbiome/metabolome, and clinical outcomes in pediatric oncology.

### 3.3. Micronutrient Status at Cancer Diagnosis: Do We Supplement?

Dr Raquel Revuelta Iniesta (UK/Spain) reviewed the current evidence on micronutrient status in CYP undergoing cancer treatment. Three systematic reviews have evaluated vitamin D in pediatric oncology [26,27,28]; all reported that deficiency was highly prevalent at diagnosis and often worsened during treatment. The latest review also suggested possible links to poorer treatment response, lower survival, and higher infection risk, although evidence has remained limited and heterogeneous due to differences in vitamin D measurement methods, as well as variations in study designs, outcome measures, and populations [28].

Beyond vitamin D, ten observational studies (*n* = 1229) reported that 96% of CYP had at least one micronutrient abnormality [29,30,31]. Prevalence at diagnosis varied widely, as most common were vitamin A, vitamin B12, folate, vitamin E, copper, magnesium, selenium, and zinc deficiencies that persisted during treatment. Patterns differed geographically; LMICs showed higher deficiencies in vitamins A and B12, folate, and zinc reflecting greater undernutrition, while deficiencies in HICs were more common in healthy-weight and overweight/obese CYP because undernourished children more often received micronutrient-enriched nutritional support. Those not receiving support frequently consumed high-energy, low-micronutrient-density diets [31]. Despite these differences, selenium and folate deficiencies have remained common globally. Importantly, few studies accounted for inflammation, medications that can affect micronutrient metabolism (e.g., *methotrexate*), or blood transfusions; thus, micronutrient status should be interpreted with caution. Folate deficiency was consistently associated with higher risks of treatment-related complications, including febrile neutropenia, neutropenia, and thrombocytopenia. However, in this systematic review no micronutrient showed a significant association with event-free survival in an unpublished meta-analysis [32] (currently under review by journal).

In the absence of randomized trials and clear dosing guidance, current recommendations have been to follow national DRVs/DRIs, monitor status throughout treatment, and correct confirmed deficiencies using pediatric guidelines. Biomarker interpretation should account for treatment-related factors and inflammation, while inflammation-sensitive markers should be assessed with CRP/albumin, and then replaced with inflammation-independent biomarkers or repeated once inflammation has resolved [33].

## 4. Rehabilitation and Prehabilitation: Integration of Nutrition and Rehabilitation in Pediatric Cancer Care

*Fit to Fight: Empowering Young Cancer Warriors Through Movement and Nutrition*

Ms Anna Gonzales (USA), part of the SIOP Rehabilitation and Physical Medicine Working Group, emphasized the importance of integrating nutrition and rehabilitation in pediatric cancer care. Research has highlighted the critical need for multidisciplinary teamwork, evidence-based guidelines, and a lifespan approach to support patients from diagnosis to survivorship [34,35]. Lifespan approaches indicated that rehabilitation depended on several factors, e.g., *etiology and severity* of the child’s health at a certain time that will determine what a child needs to ensure they achieve high-level function to improve quality of life [36].

The guiding principle was to enable CYP with cancer to maintain normal childhood activities and quality of life by bridging nutritional support and physical rehabilitation, recognizing their combined impact on outcomes. This work underlined nutrition as a modifiable factor, essential for optimal care and improved results in pediatric oncology [37,38,39].

## 5. Assessment Tools and Guidelines

### 5.1. Nutritional Guideline Development for High-Income Countries (HICs)

The existing literature consistently demonstrated the prognostic significance of nutritional status in childhood cancer. However, variability in the timing and frequency of nutritional assessments, the delivery of nutrition interventions, and the accurate diagnosis of malnutrition has continued to impede high-quality clinical nutrition care, as highlighted by Dr Amy Lovell (New Zealand) [40,41,42,43,44]. Implementing guidelines or models of care can support the standardization of practice, reduce unwarranted variation, enhance translation of evidence into clinical settings, and improve patient care, safety, and outcomes [45].

Currently, no international nutrition guidelines exist for CYP with a cancer diagnosis. One of the priority projects for HICs of the SIOP Nutrition Network over the next three years is being co-led by Dr Lovell and Dr Revuelta-Iniesta (UK/Spain). They have convened an international transdisciplinary group that will collaborate with the research group in New Zealand and develop evidence-based nutrition guidelines for CYP affected by childhood cancer in HICs.

To achieve this, the team has generated a comprehensive list of 30 clinical questions covering major domains of pediatric oncology nutrition. These questions are currently undergoing a structured prioritization exercise with international clinicians, researchers, and consumer representatives. Because this process is ongoing, the full list of technical questions has not been listed here; however, the prioritized questions will guide evidence reviews and syntheses that will underpin guideline recommendations.

The guideline development process will follow the *Handbook for Guideline Development* [46] including: (1) determining the scope, (2) prioritizing clinical questions, (3) synthesizing the available evidence for each question, (4) appraising the certainty of the evidence, and (5) developing actionable recommendations in process of preparing clinical practice guidelines.

### 5.2. Validation of Mid-Upper Arm Circumference as an Assessment Tool in Childhood Cancer

Children with cancer have frequently experienced undernutrition, especially those over five years old, for whom international assessment standards have been lacking [11,47]. Dr Judy Schoeman (South Africa) summarized recent efforts to update and validate anthropometric measurement guidelines for older children [48], highlighting the use of country-specific cutoffs for mid-upper arm circumferences (MUAC) [49] and emphasizing that Z-scores have correlated better with nutritional status than fixed cutoff values [50]. MUAC has been particularly useful in the field of pediatric oncology, as it is a quick measurement as well as a more sensitive and reliable indicator of nutritional status, as compared to BMI or WFH, which can be misleading due to factors such as tumor mass and fluid retention [51]. MUAC has also been particularly useful and practical in busy clinics due to time constraints and limited resources, while cutoff values can simplify the process of identifying children as undernourished.

In 2020, the SIOP Nutrition Network, as representatives of the pediatric oncology [51] community, released WHO-based guidelines (2006 standards) [52] on the anthropometric assessment of a child with cancer, tailored to institutional capacity that determined the levels of care provided [53]. The Nutrition Network steering committee is currently undertaking a comprehensive review process to determine whether updated recommendations can be formulated for MUAC older than five years of age, or whether the SIOP 2020 guidelines remain the most appropriate standard given the current evidence [53]. The final recommendations are anticipated and will be published in coming months (manuscript under review).

### 5.3. ARIA Guide: Nutrition Portal

The Adapted Resource and Implementation Application (ARIA) guide is a comprehensive, globally collaborative initiative that has developed a web-based clinical decision aid tool and dissemination platform to facilitate access to resource-stratified, evidence-based clinical practice recommendations. The executive sponsors for ARIA guide are SIOP and St. Jude Global, and the SIOP Nutrition Network has been a key partner in developing the ARIA guide nutrition portal. Ms Liz Sniderman (Canada) summarized the methodology, structure, and implementation strategy of the ARIA guide nutrition portal, which has aimed to facilitate access to resource-stratified nutrition guidance for pediatric oncology healthcare providers worldwide. The nutrition portal will include comprehensive, resource-adapted clinical practice recommendations for the assessment and management of malnutrition in children with cancer, as this portal has been developed with recommendations from global expertise (HICs and LMICs), has taken into account resource availability, and will assist teams to identify malnutrition in a few seconds with a interactive nutritional status assessment tool.

Through a rigorous methodology incorporating systematic literature reviews, critical appraisal, expert consensus, and contextual adaptation, the ARIA guide can create living guidance documents that can remain responsive to emerging evidence. This work has represented a significant step forward in democratizing access to nutrition knowledge in pediatric oncology and supporting equitable care delivery across diverse resource settings globally [54].

## 6. Building Global Communities of Practice and Global Research Initiatives and Capacity Building

### 6.1. IIPAN/IARC/EPICkids

Prof Elean Ladas, the founder of IIPAN at Columbia University Irving Medical Centre, presented a synopsis of their clinical guidelines for LMICs [55], as well as a comprehensive overview of locally driven, evidence-based nutritional studies being performed across multiple global regions leveraging the IIPAN research framework [56]. The IIPAN research portfolio has been advancing nutritional science related to the cost of care, body composition, micronutrient interventions, dietary interventions, and food-based therapeutic approaches in children with cancer. In collaboration with the International Agency for Research on Cancer (IARC) and the World Health Organization (WHO), IIPAN and EPICkids are co-leading a multinational microbiome/metabolome study on clinical outcomes in children with ALL and brain tumors. Recruitment for this study started in June 2023 and will continue through to December 2028, aiming to recruit 900 patients with ALL and 1400 patients with a brain tumor—the study is expected to be completed in December 2031. EPICkids primary aims have been to identify personalized nutritional pathways that may enable targeted nutritional interventions to further improve outcomes in pediatric cancer. Collectively, this systematic approach has demonstrated the power of global collaborations to answer complex nutritional questions by examining the underlying mechanisms of nutritional status and clinical outcomes in childhood cancer [57].

### 6.2. ICONIC Formation and Development of WHO Knowledge Action Portal

Prof Stephen Wootton (UK) represented the International Collaboration on Nutrition in Cancer (ICONIC) as their focus lies on building an international community of practice to improve nutritional care for CYP with cancer, emphasizing the pressing need for collaboration and knowledge-sharing across professional and geographic boundaries. Despite longstanding evidence supporting the pivotal role of nutrition in cancer care, its integration into clinical practice has often remained incomplete; he highlighted barriers such as limited awareness among healthcare professionals outside nutrition circles and the unique nutritional risks facing young adults—a group frequently overlooked between pediatric and adult services.

He described the formation of ICONIC, which was established through partnerships with leading organizations including the International Union of Nutritional Sciences (IUNS), the National Institute for Health and Care Excellence (NIHR) in the UK, and the WHO. ICONIC has provided openness and inclusivity, inviting participation from patients, families, clinicians, researchers, and policy advocates, regardless of formal memberships or fee barriers [58].

A significant innovation described was the use of the WHO Knowledge Action Portal, a knowledge-sharing community platform hosting resources, mapping, collaboration tools, and open-access directories for global participation. This platform has been critical for sharing both scientific evidence and experiential insights, especially from settings with limited formal research infrastructure. Prof Wootton stressed that practical workplace experiences, such as the development of the WHO’s protocol for the management of severe acute malnutrition, can be as valuable as systematic reviews, and the community should embrace both [59].

The presentation also addressed new research priorities, such as the management of post-treatment weight gain, and flagged the need to balance short-term recovery goals with long-term health risks, such as cardiovascular disease. Prof Wootton concluded with a call for the harmonization of clinical practices, active consensus building, and the extension of global networks so nutritional care standards can benefit all CYP affected by cancer everywhere.

## 7. Beyond the Proposal: Strategizing for Impact in Grant Writing—A WCRF International Perspective

Dr Julia Panina from the World Cancer Research Fund (WCRF) presented essential strategies for developing competitive grant proposals in nutrition and cancer research. These principles can be directly applicable to pediatric oncology nutrition studies seeking funding to improve outcomes for children with cancer.

She spoke about the nine critical elements for a successful grant application, including funder alignment; novelty; research environment; scientific rigor; feasibility; patient and public involvement; clarity; budget justification; and impact beyond publications.

She also highlighted the current prioritized areas of the WCRF, including environmental contaminants in food/water; artificial intelligence in research and healthcare; microbiomes; rare childhood cancers; lifestyle improvements in treatment responses; ultra-processed food and specific food (e.g., *sugary drinks, tea, coffee, soy*) diets; and sedentary behavior.

Grant funding opportunities were mentioned, including regular, feasibility, and early career grants. Lastly, she emphasized the importance of building relationships with funders to support success and translate research into real-world outcomes [60].

## 8. Oral Abstract Presentations by Delegates

Ms Nora Lara Pompa (Mexico) presented research about enteral nutrition as a key component of nutritional management, though its prescription has remained non-standardized, particularly in the case of blenderized enteral nutrition. This retrospective study described blenderized nutrition prescription and its associations to short-term clinical outcomes in children with cancer (any type) (0–18 years) admitted to the intensive care unit at a tertiary pediatric oncology hospital. These feeds were associated with shorter lengths of stay and days on mechanical ventilation. She proposed future prospective studies to explore these associations and their outcomes and to clarify different aspects of blenderized prescription in this population [61].

Ms Ruijie Li (UK) presented findings from the Swiss CardioOnco study, a multicenter cohort of adult survivors of childhood cancer in Switzerland, which examined lifestyle behaviors in relation to cardiotoxic cancer treatments received during childhood. The analysis showed that the overall adherence to recommended health behaviors was generally low among survivors. Importantly, no significant differences were observed in cardiovascular disease prevalence or lifestyle behaviors across cardiotoxic treatment risk groups. These findings highlighted that childhood cancer survivors with cardiovascular risks need universal health promotion strategies focusing on diet, physical activity, smoking cessation, and alcohol moderation across the entire childhood cancer survivor population [61].

Ms Debbi Rowley (UK) discussed the potential role for prehabilitation in HSCT patients. The scoping review she and her team performed identified 137 studies, of which nine were included in the review, but mostly low-quality data was found with hardly any nutritional and physical function interventions were presented; however, these findings suggested that early physical and nutritional intervention could improve outcomes. This has emphasized the need for further multi-modal, multicenter research, as well as supporting grant applications for intervention protocols [61].

Dr Emilie Bertrand (New Zealand) shared results from a prospective cohort study on symptom burden and diet quality in children with cancer. This was measured at diagnosis and then three, six, nine and twelve months after diagnosis. They found that, although symptom burden declined over time, its impact on distress, diet quality, and malnutrition risk persisted and influenced long-term health. Therefore, proactive symptom management and individualized nutritional support is essential to mitigating the effects of nausea, pain, and fatigue on dietary intake [61].

## 9. Round Table Discussions

The delegates were divided into three groups according to their experience in the field and/or their interest in discussing nutrition-related research goals for the next five years:

### 9.1. Prehabilitation Strategies

The group agreed on the need to strengthen international, multidisciplinary, and multicenter efforts to advance prehabilitation research in pediatric oncology. Prehabilitation was defined as a multimodal preventive intervention designed to enhance a patient’s physical, nutritional, and psychological resilience before and during cancer treatment, with the aim of improving treatment tolerance, reducing complications, and accelerating recovery [62]. The group emphasized the importance of co-designing future studies across disciplines (*nutrition, physiotherapy, pediatric oncology and psychology*) and centers to ensure feasibility and scalability. Priority research goals included determining the optimal timing for initiating prehabilitation, integrating nutritional interventions along the cancer treatment pathway, and harmonizing the assessment of core outcomes across studies, including quality of life, treatment tolerance, nutritional status, and prognosis.

### 9.2. Nutritional Status and Pharmacology

The gap between evidence-based practice and evidence-informed practice in pediatric chemotherapy administration with regard to nutritional status, especially moderate-to-severe malnutrition as well as obesity, was highlighted. Current treatment guidelines and protocols do not adequately address severe undernutrition or obesity, both of which have been highly prevalent in various regions worldwide. Recent reviews have highlighted this gap and underscored the need to incorporate nutritional status into future guidelines and studies. The group also emphasized the importance of pharmacokinetic research in LMICs, where up to 60% of children with cancer can be malnourished. A first step could be a feasibility study evaluating historical chemotherapy drugs in populations outside the USA and Europe. Furthermore, the group stressed the need for advocacy, including engagement with clinical trial groups and pharmaceutical companies, to ensure that newly developed drugs were also studied and optimized for nutritionally vulnerable populations.

### 9.3. Advocacy

The session highlighted systemic barriers to nutrition integration in cancer care and the need for proposed actions/strategies for improvement. The barriers included integration of childhood cancer as a specific risk factor in the WHO nutritional guidelines, lack of professional recognition for dietitians, minimal nutrition representation in cancer care organizations, policies, and critical workforce, and training gaps, especially in LMICs. Proposed actions for successful advocacy would require liaising with the SIOP Advocacy Group and SIOP Advocacy Committee to ensure that nutrition is recognized and prioritized within SIOP initiatives, as well as persistent, evidence-based strategies such as engaging regional societies, securing nutrition lecture slots at conferences with representatives on the Scientific Committee, and collaborating with ministries around the globe. The need for accessible training for dieticians, including nutrition in policies and cultural adaptation, had been highlighted to improve nutritional care and outcomes for children with cancer worldwide.

## 10. Discussion

The first SIOP Nutrition Network Research Forum marked a significant milestone in advancing nutrition as a core component of pediatric oncology care and research. By bringing together international experts from HICs and LMICs, the forum provided a unique platform to synthesize current evidence, critically examine persistent knowledge gaps, and align global research priorities across disciplines. The presentations highlighted emerging evidence on body composition and treatment outcomes, the role of the microbiome and micronutrients, the integration of nutrition within rehabilitation and prehabilitation frameworks, while also identifying substantial gaps in high-quality interventional research and standardized assessment approaches alongside a continuous call for the integration of nutritional care into global pediatric oncology guidelines.

Through focused roundtable discussions on prehabilitation, pharmacology and drug dosing, and advocacy, delegates reached consensus on the urgent need for coordinated, international, multidisciplinary, and multi-center efforts. In particular, there was strong agreement that prehabilitation represented a key opportunity to improve treatment tolerance, reduce complications, and enhance recovery, provided that future studies adopted co-designed approaches, harmonized outcomes, and used robust methodologies. Of equal importance, discussions on pharmacokinetics emphasized the clinical implications of body composition for drug dosing, while advocacy sessions highlighted systemic barriers to nutrition integration and the need for workforce development, policy engagement, and global capacity building.

Following the meeting, an online post-forum survey was sent to all delegates with questions about the presentation from speakers, roundtable discussions, the venue, and the impact of the day. The results (79% response) indicated a high satisfaction score (overall 9.1/10), demonstrating that the forum had a strong impact and set a model for future pediatric oncology nutrition research meetings.

## 11. Conclusions

The forum established the first internationally endorsed up-to-date roadmap for pediatric oncology nutrition research, fostering collaboration across regions and resource settings. The priorities and partnerships emerging from this meeting provide the foundation for generating high-quality evidence, accelerating the translation of research into practice and strengthening the recognition of nutrition as an essential, modifiable determinant of outcomes for CYP with cancer worldwide. The Advocacy Group has aimed to liaise closely with the SIOP Advocacy Committee to ensure that nutrition is recognized and prioritized within SIOP initiatives. In addition, the group has planned to develop a position statement for the WHO highlighting the critical role of nutrition in pediatric oncology care. A key objective of the forum over the next two years will be to develop SIOP-led research protocols and seek funding to support the activities of the Rehabilitation and Pharmacokinetics Group. In parallel, we have aimed to establish a global consortium to undertake pilot studies that will inform the design of larger international collaborative trials. By harmonizing methodologies and pooling data across centers, these efforts have the potential to generate high-quality evidence and drive meaningful improvements in outcomes for children with cancer.

## Data Availability

No additional data available.

## Supplementary Materials

The following supporting information can be downloaded at: https://www.mdpi.com/article/10.3390/nu18071112/s1; Table S1: Contact details of all delegates who attended the SIOP Nutrition Research Forum Meeting (excluding the authors) on 24 October 2025 and had input in different sections of the manuscript is available.

## Abbreviations

The following abbreviations are used in this manuscript:

aGvHD	Acute Graft-Versus-Host Disease
ALL	Acute Lymphoblastic Leukemia
ARIA	Adapted Resource and Implementation Application
BIA	Bioelectrical Impedance Analysis
BMI	Body Mass Index
CI	Confidence Interval
CRP	C-reactive Protein
CT	Computed Tomography
CVD	Cardiovascular Disease
CYP	Children and Young People
DEXA	Dual-energy X-ray Absorptiometry
DLW	Doubly Labeled Water
DRI	Dietary Reference Intake
DRV	Dietary Reference Value
EFS	Event-Free Survival
ESPGHAN	European Society for Paediatric Gastroenterology, Hepatology and Nutrition
HIC	High-Income Country
HSCT	Hematopoietic Stem Cell Transplantation
IAEA	International Atomic Energy Agency
ICONIC	International Collaboration on Nutrition in Cancer
IIPAN	International Initiative for Pediatrics and Nutrition
IUNS	International Union of Nutritional Sciences
LMIC	Low- and Middle-Income Country
MUAC	Mid-Upper Arm Circumference
NIHR	National Institute for Health and Care Research
OS	Overall Survival
RCT	Randomized Controlled Trial
SIOP	International Society of Pediatric Oncology
WHO	World Health Organization
WCRF	World Cancer Research Fund
