# Supplementary material for: Establish Global Nutrition Research Strategies: The Meeting Report of the First SIOP Nutrition Research Forum
Source: Nutrients. 2026 Mar 30;18(7):1112. doi: 10.3390/nu18071112 (PMC13075039; doi:10.3390/nu18071112)
Supplement: Supplementary file 1 [file nutrients-18-01112-s001.zip › nutrients-4173033-supplementary.pdf]

Supplementary Table S1: Delegates attended the SIOP Nutrition Research Forum on 24 October 2025, Utrecht, the Netherlands

Name	Surname	Designation	Affiliation	City & Country	Email address
Adrian	Martyniak	Research assistant	University Children's Hospital of Cracow/Jagiellonian University Medical College	Krakow, Poland	adrian.martyniak@uj.edu.pl
Aleksandra	Stanisławska	Doctor	University Clinical Center Gdańsk	Gdańsk, Poland	aleksandra.stanislaska@uck.gda.pl.
Alexia	Murphy-Alford	Nutritionist	International Atomic Energy Agency	Vienna, Austria	A.Alford@iaea.org
Alexis	Ross	Dietitian	University of Auckland	Auckland, New Zealand	alexis.ross@auckland.ac.nz
Amanda	Galvin	Dietitian	Children's Health Ireland	Dublin, Ireland	amanda.galvin@childrenshealthireland.ie
Anna	Gonzales	Physical Therapist	Nationwide Children's Hospital	Columbus, Ohio USA	Anne.Gonzales@nationwidechildrens.org
Breeana Louise	Gardiner	Dietitian	Great Ormond Street Hospital for Children	London, UK	Breeana.gardiner@gosh.nhs.uk
Christina	Katsagoni	Nutritionist	IIPAN/Aghia Sofia Children's Hospital	Athens, Greece	christina.katsagoni@gmail.com
Deborah	Rowlley	Paediatric Physiotherapist	Sheffield Children's FT	Sheffield, Uk	deborah.rowley2@nhs.net
Dieuwertje	Kok	Dietitian	Wageningen University	Wageningen, Netherlands	dieuwertje.kok@wur.nl
Douglas	Momberg	Senior Researcher - biotechnology	University of the Witwatersrand	Johannesburg, South Africa	Douglas.Momberg@wits.ac.za
Elena	Ladas	Dietician	Columbia University	New York, USA	ejd14@cumc.columbia.edu
Emanuel Alex	Nkya	Nutritionist	KCMC hospital /IIPAN	Kilimanjaro, Tanzania	emanuelalex935@yahoo.com
Émilie	Bertrand	Exercise Physiologist	The University of Auckland	Auckland, New Zeeland	emilie.bertrand@auckland.ac.nz
Erika	Damasco	Dietician	IIPAN / Columbia University Irving Medical Center	New York, USA	ed3037@cumc.columbia.edu
Evan	Munoz	Nutritionist	IIPAN/Niño Jesus/La Paz	Madrid, Spain	munoz.evan@protonmail.com

Eveline	van den Heuvel	Dietitian	Starship Children's Hospital NZ	Auckland, New Zealand	thefourhills@xtra.co.nz
Fabien	Belle	Nutritionist	Institute of Social and Preventive Medicine, University of Bern	Bern, Switzerland	fabien.belle@unibe.ch
Gareth	Veal	Pharmacist	Newcastle University Centre for Cancer	Newcastle Upon Tyne, UK	gareth.veal@newcastle.ac.uk
Giorgia	Preziati	Nutritionist	Fondazione IRCCS Istituto Nazionale Tumori	Milan, Italy	Giorgia.Preziati@istitutotumori.mi.it
Janne Anita	Kvammen	Dietitian	Oslo University Hospital	Oslo, Norway	uxjaam@ous-hf.no
Jessie	Sinkhonde	Nutritionist	Baylor Malawi	Malawi	sinkhonde@baylor-malawi.org
José	van Tongeren	Dietitian	Prinses Máxima Centrum	Wijk bij Duurstede, Nederland	dietetiek-3@prinsesmaximacentrum.nl
Julia	Panina	Research funding	World Cancer Research Fund International	London, UK	j.panina@wcrf.org
Karina	Viani	Dietician	IIPAN, FMUSP	São Paulo, Brazil	viani.karina@gmail.com
KC	Anand	Paediatric Oncologist	Sri Shankara Cancer Hospital and Research Centre, Bengaluru	Bengaluru, India	kcanand2006@gmail.com
Kristin	Mellett	Dietitian	Moansh Children's Hospital	Victoria, Australia	kristin.mellett@monashhealth.org
Kristina	Petrova	Pediatric oncologist	University hospital Sveta Marina	Varna, Bulgaria	petrova_kris@yahoo.com
Laura	Sealy	Dietitian	University Hospitals Bristol & Weston NHS Trust	Bristol, UK	Laura.Sealy@uhbw.nhs.uk
Lisanne	Renting	Nutritionist	Prinses Máxima Centrum	Utrecht, Nederland	l.renting@prinsesmaximacentrum.nl
Liz	Sniderman	Nurse Practitioner	St. Jude Children's Research Hospital	Memphis, USA	Liz.Sniderman@stjude.org
louise	Henry	Dietician	Royal marsden Hospital	London, UK	louise.henry@rmh.nhs.uk
Marciel	Pedro	Paediatric Oncologist	Philippine General Hospital	Manila, Philippines	info@the-cancer-foundation.org
Mariana	Kruger	Paediatric Oncologist	Stellenbosch University	Stellenbosch, RSA	marianakruger@sun.ac.za

Marta	Llopis Lera	Nutritionist	Hospital Sant Joan de Déu/IIPAN	Barcelona, Spain	marta.llopis@sjd.es
Monika	Ziętarska	Dietitian	University Clinical Center in Gdańsk	Gdańsk, Poland	monika.zietarska@uck.gda.pl
Nara Elizabeth	Lara Pompa	Dietitian	Hospital Infantil Teleton de Oncología	Queretaro, Mexico	nara.lara@hospitalteleton.org.mx
Nina	van der Linden	Dietitian	Prinsess Maxima Center	Utrecht, Netherlands	n.c.vanderlinden-4@prinsesmaximacentrum.nl
Pia	Leon	Dietitian	Sanatorio aleman	Concepción,Chile	Pialeon2013@gmail.com
Rita	Haukkovaara	Dietitian	New Children's hospital, Helsinki University Hospital	Helsinki, Finland	Rita.haukkovaara@**helsinki**.fi
Ruijie	Li	**Exercise physiologist**	University of Exeter	Exeter, UK	rl615@exeter.ac.uk
Sofia	Rizzari	Nutritionist	Fondazione IRCCS San Gerardo dei Tintori Monza	Monza, Italy	sofia.rizzari@irccs-sangerardo.it
Sofie	van der Zalm	Dietician	Wageningen University & Research	Wageningen, Netherlands	sofie.vanderzalm@wur.nl
Stephen	Wootton	Dietician	University Hospital Southampton	Southampton, UK	s.a.wootton@soton.ac.uk
Umme	Ali	Dietitian	Addenbrooke's Hospital	Cambridge, UK	ali.umme@pals@nhs.net
Vicki	Clarke	Dietitian	Christchurch Hospital	Christchurch, New Zealand	Vicki.Clarke@cdhb.health.nz
Wieger	Voskuijl	Pediatrician	Amsterdam UMC	Amsterdam, Netherlands	w.p.voskuijl@amsterdamumc.nl
Wim	Tissing	Paediatric Oncologist	Princess Maxima Center	Utrecht, Netherlands	w.j.e.tissing@prinsesmaximacentrum.nl
